# Random protein sequences can form defined secondary structures and are well-tolerated *in vivo*

**DOI:** 10.1038/s41598-017-15635-8

**Published:** 2017-11-13

**Authors:** Vyacheslav Tretyachenko, Jiří Vymětal, Lucie Bednárová, Vladimír Kopecký, Kateřina Hofbauerová, Helena Jindrová, Martin Hubálek, Radko Souček, Jan Konvalinka, Jiří Vondrášek, Klára Hlouchová

**Affiliations:** 1 Department of Biochemistry, Faculty of Science, Charles University, Hlavova 2030, 128 00 Prague 2, Czech Republic; 2 Institute of Organic Chemistry and Biochemistry, Czech Academy of Sciences, Flemingovo náměstí 2, 166 10 Prague 6, Czech Republic; 3 Institute of Physics, Faculty of Mathematics and Physics, Charles University, Ke Karlovu 5, 121 16 Prague 2, Czech Republic

## Abstract

The protein sequences found in nature represent a tiny fraction of the potential sequences that could be constructed from the 20-amino-acid alphabet. To help define the properties that shaped proteins to stand out from the space of possible alternatives, we conducted a systematic computational and experimental exploration of random (unevolved) sequences in comparison with biological proteins. In our study, combinations of secondary structure, disorder, and aggregation predictions are accompanied by experimental characterization of selected proteins. We found that the overall secondary structure and physicochemical properties of random and biological sequences are very similar. Moreover, random sequences can be well-tolerated by living cells. Contrary to early hypotheses about the toxicity of random and disordered proteins, we found that random sequences with high disorder have low aggregation propensity (unlike random sequences with high structural content) and were particularly well-tolerated. This direct structure content/aggregation propensity dependence differentiates random and biological proteins. Our study indicates that while random sequences can be both structured and disordered, the properties of the latter make them better suited as progenitors (in both *in vivo* and *in vitro* settings) for further evolution of complex, soluble, three-dimensional scaffolds that can perform specific biochemical tasks.

## Introduction

The proteinogenic amino acid alphabet has remained largely unchanged during the past ~3 billion years of astonishing evolutionary diversification. The 20 amino acid building blocks could be combined to construct a plethora of polypeptides, yet only a fraction of potential sequences are found in life on Earth^1,2^. For example, 10^130^ possible sequences for a 100-residue polypeptide can be formed from the canonical alphabet, but the number of existing proteins is estimated to be at most 10^15^ ^1^.

It appears that a finite, relatively small library of protein domains has evolved^3–5^. Structural classification databases (such as SCOP and CATH) have amassed ~1,500 different domain families that account for more than 70% of genomic sequences^6–8^. The prevailing assumption is that once evolution arrived at a set of stable protein folds, evolutionary pressure was dominated by functional constraints^9^. In most cases, a protein’s structure determines its functional properties. This raises the question of whether a defined secondary or tertiary structure is a unique property of the sequences found in nature, or whether random sequences also have the potential to form defined structures. Understanding how the structural potential of natural protein sequences differs from that of sequences not subjected to billions of years of evolutionary constraints could provide insights into evolutionary history.

Contrary to early assumptions, a few recent studies suggest that there are unknown functional folds outside the natural protein space, but estimates of their frequency differ^10–12^. Systematic characterization of the folding potential of random sequences has been attempted using tertiary structure prediction algorithms such as Rosetta, but parallel studies questioned the reliability of these algorithms for random sequences unrelated to those found in nature^13,14^. In their Rosetta *ab initio* study, Minervini *et al*. reported that random sequences (with equal relative content of each amino acid) have higher α-helical content (by nearly 10%) and lower β-sheet content than biological sequences. Using a single predictor of secondary structure occurrence, Yu *et al*. recently reported an opposite conclusion of the α-helical/β-sheet preference, although they used nearly the same input parameters as Minervini *et al*.^15^. Besides a random dataset with equal relative content of each amino acid, Yu *et al*. additionally included a random dataset with natural occurrence of amino acids, both reporting a comparable distribution of secondary structure content. A few experimental studies have used random 50- to 80-residue sequences to assess secondary structure outside the natural protein space, but these studies had to rely on relatively sparse sampling^2,16,17^. The researchers estimated that compact folding is a property for 5–20% of random sequences^16,17^. Taken together, all of these studies agree that formation of secondary and tertiary structures seem to be general features of polypeptide chains. However, there is a clear lack of correlation among the available bioinformatics/experimental studies, making it difficult to draw conclusions about protein structure evolution.

Here, we present a systematic computational and experimental exploration of the amino acid alphabet and the structural and biophysical consequences of random sequence formation. We generated an *in silico* library (10^4^ sequences) of 100-amino-acid proteins and evaluated the occurrence of secondary structure by 5 different prediction algorithms, comparing the properties of random polypeptides with those of natural proteins. Next, we selected 3 × 15 candidate proteins from the library based on their predicted properties (high, low, or random secondary structure occurrence) and experimentally characterized the individual proteins. Because they stem from identical input parameters, the outcomes of these two approaches can be directly compared, allowing us to assess the prediction algorithm accuracy when applied to the unevolved sequence space.

## Results and Discussion

### Frequencies of secondary structure motifs are similar in random sequences and biological proteins

We used numerous bioinformatic predictors of secondary structure and protein disorder to compare four polypeptide libraries: (A) **random** sequences in which the ratios of individual amino acids reflect those found in natural proteins, (B) fragments of natural proteins from the TOP8000 database of non-redundant structurally characterized proteins extracted from the **PDB** database, (C) a selection of fragments of natural proteins from the **Uni**Prot database, and (D) fragments of natural intrinsically disordered proteins (IDPs) from the **Dis**Prot database^18–20^. The four libraries each comprise 10^4^ 100-residue sequences (the predictions were performed with the same outcome also with 109-residue sequences including additional residues that were added for the purpose of recombinant expression). Additionally, we investigated the similarity of the random and characterized protein sequences. Only low-significant matches were found by BLAST method for the whole set (Fig. S1) as well as for sequences chosen for experimental characterization (Table S1).

**Figure 1:**
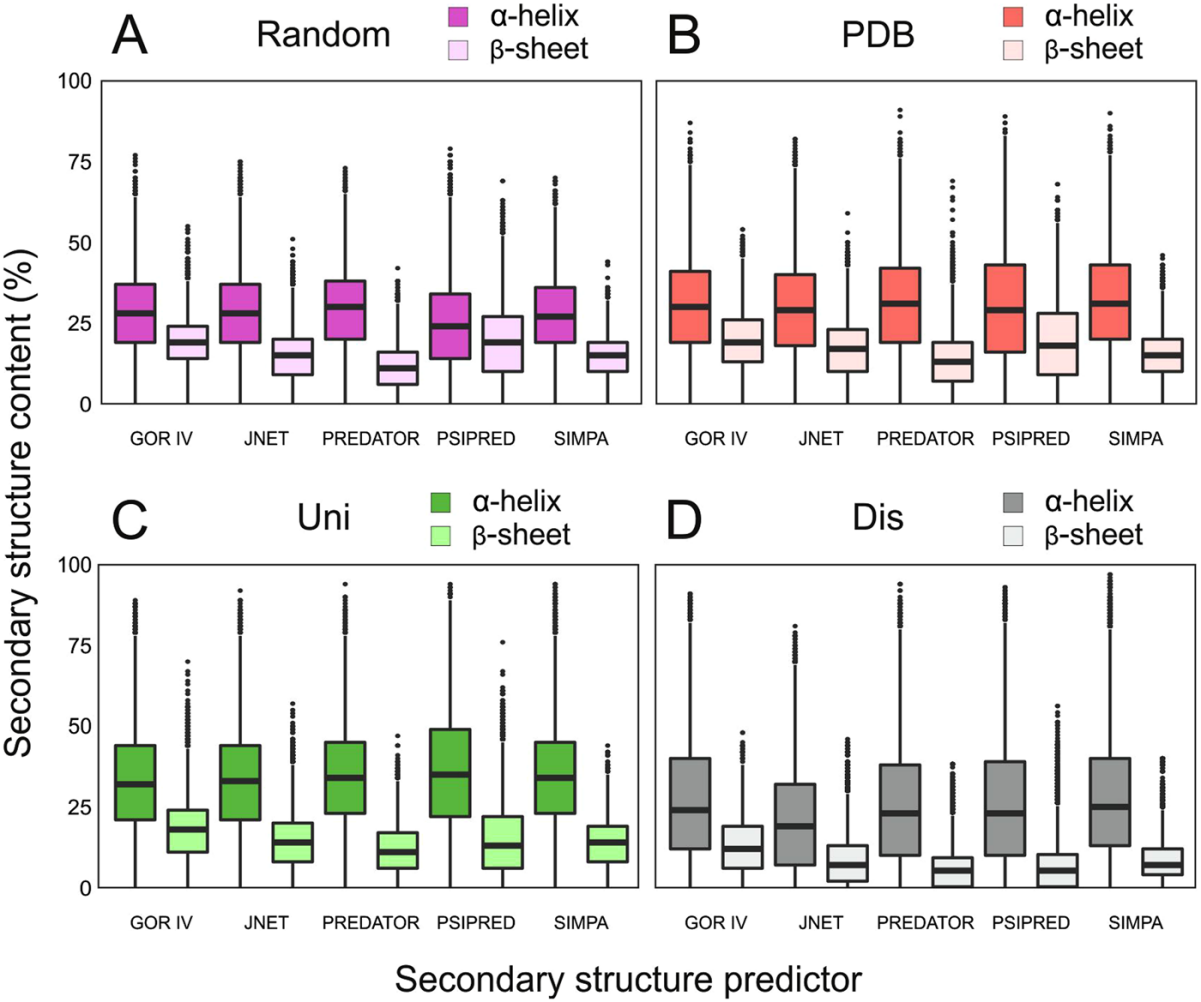
Predictions of secondary structure occurrence in the (**A**) random, (**B**) PDB, (**C**) Uni, and (**D**) Dis libraries. α-helical and β-sheet content determined by five different predictors are shown with statistical information. The center of the box represents the median, and the upper and lower borders represent the 3rd and 1st quartile, respectively. The solid lines illustrate the maximal value and minimal value, excluding outliers, which are shown as dots. The Dis dataset secondary structure prediction is included as a negative reference.

According to statistical analyses of the bioinformatic predictions, both the overall occurrence of secondary structure and the distribution of motifs were comparable for the random and Uni/PDB protein sequence space (Fig. 1 and Table S2). The total occurrence of secondary structure motifs was approximately 5% lower for the random sequence library than for the Uni/PDB natural protein datasets (Table S2). Therefore, our results did not identify any profound differences between random and biological sequences secondary structure formation and thus contrast with previous reports that were based on a single secondary or tertiary structure prediction and which reported statistically significant differences^13,15^. The overall α-helical and β-sheet content predicted by the different algorithms correlate well for all libraries in our study, with an average Pearson correlation coefficient of approximately 0.7 (Table S3).

### Experimental sampling of random sequences confirms frequent occurrence of secondary structure and demonstrates tolerance *in vivo*

**Figure 2:**
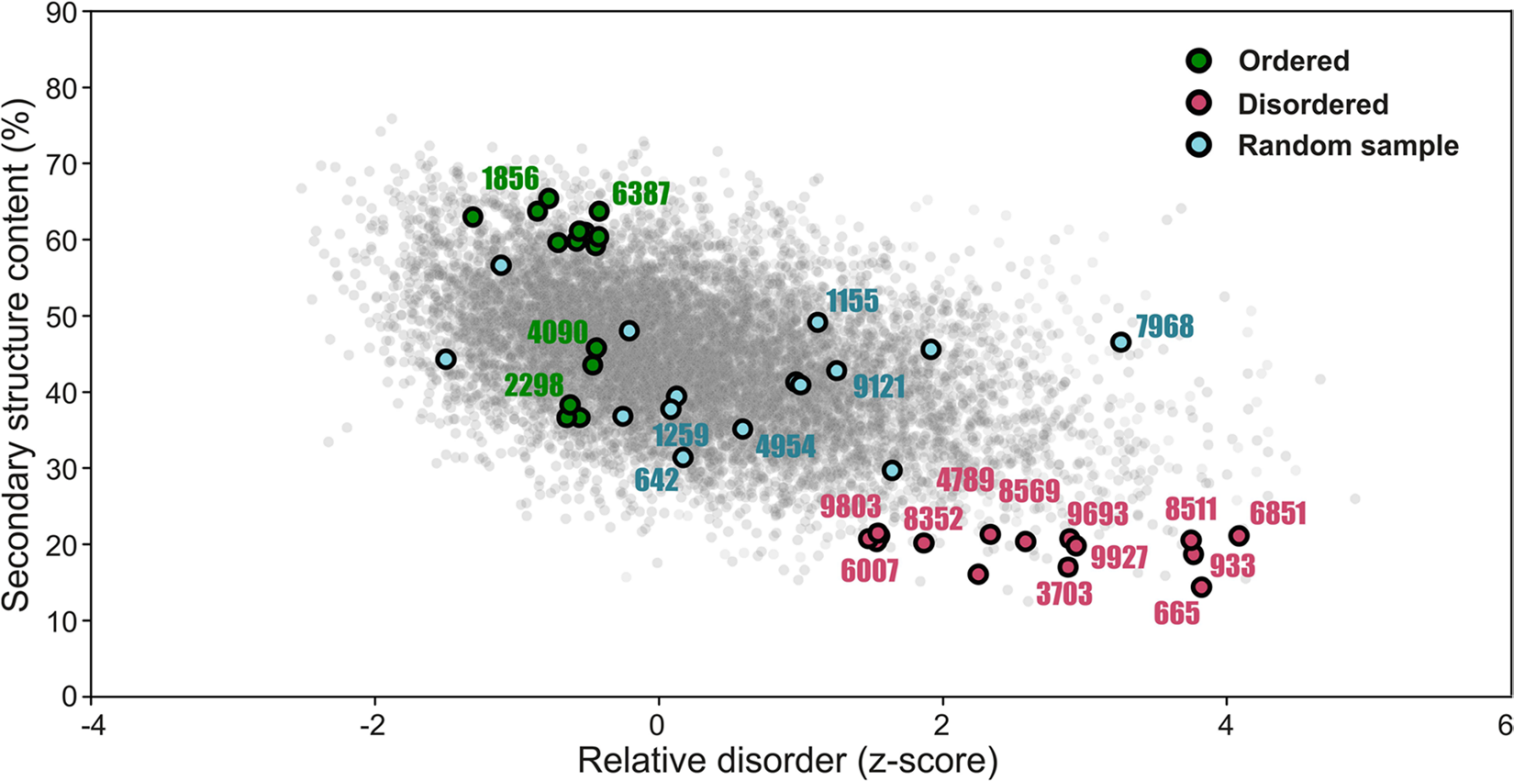
Selection of sequences from the random dataset for experimental characterization. Scatter plot of the secondary structure (y-axis) and disorder prediction (x-axis) in which the selection of group 1 (green), 2 (blue), and 3 (red) proteins for experimental sampling is highlighted as circles (circles with 4-digit codes assigned were successfully purified and characterized). The secondary structure prediction is based on the overall average structure content stemming from five different predictors. The final disorder score is based on the z-value of the average rank of individual sequences from four different disorder predictors.

Based on the bioinformatic analyses, three groups of 15 proteins each were selected from the random sequence library based on the following criteria (Fig. 2):

GROUP 1: (i) High occurrence of predicted secondary structure (samples with both α-helices and β-sheets) and low disorder, (ii) high predicted solubility

GROUP 2: Random selection

GROUP 3: (i) Low occurrence of predicted secondary structure and high disorder, (ii) high predicted solubility

**Figure 3:**
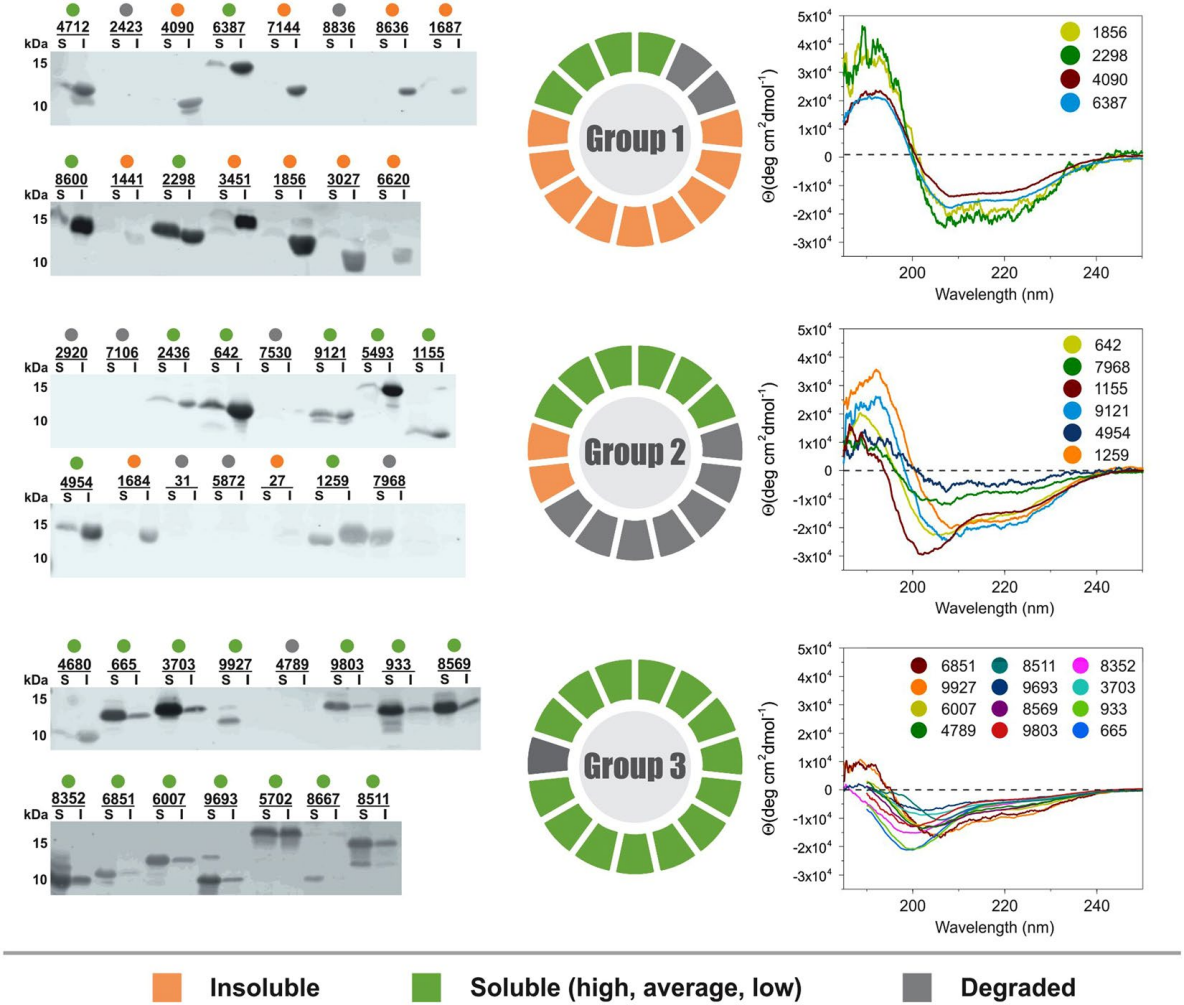
Results of experimental sampling. **Left** – Western blot solubility assay showing never-born proteins (NBPs) expressed in *E. coli* in the insoluble (I) and soluble (S) - including all high, average and low levels of solubility - fractions; **Middle** – a pie graph reporting the expression profiles for group 1–3 NBPs; **Right** – electronic circular dichroism spectra of group 1–3 NBPs that were successfully overexpressed and purified.

DNA sequences encoding the selected never-born proteins (NBPs) were synthesized so that each NBP has methionine as the N-terminal residue and a 6 × His tag at the C-terminus. The bioinformatic predictions were repeated with sequences including the methionine and 6 × His tag, to confirm that there were no variations between the predictions of the unmodified and modified sequences. The NBPs were recombinantly expressed in *E. coli* BL21(DE3), and the protein expression level and solubility were analyzed. Out of 15 proteins in each group, the following expressed/soluble ratios were observed: 13/4 in group 1, 8/6 in group 2, and 14/14 in group 3 (Fig. 3). Notably, protein overexpression and solubility in cells increased from group 1 (most structured) to group 3 (least structured). In total, 22 proteins were successfully overexpressed and purified for further characterization. While group 1 proteins have pronounced ellipticity and minima between 205–220 nm in their electronic circular dichroism (ECD) spectra (typical of proteins with high secondary structure content), group 3 ECD spectra indicate proteins with low structural content (Fig. 3). Low concentration of denaturing agent (0.4 M guanidinium hydrochloride) moderately decreases ellipticity for group 1 proteins unlike for group 3 proteins. Conversely, upon addition of a helical structure inducer (50% trifluoroethanol), structure is significantly induced in group 3 spectra, indicative of its original lack of structure unlike for group 1 proteins (Fig. S2). This observation is further supported by the 1D^1^H NMR spectra (Fig. S3). The overall signal dispersion in the spectra obtained for group 1 proteins (panels A and B) suggests the presence of a hydrophobic core. In addition, the relatively broad signals are indicative of a certain degree of aggregation. The narrow and significantly less dispersed signals observed in the spectra of group 2 and 3 proteins are typical for IDPs.

**Figure 4:**
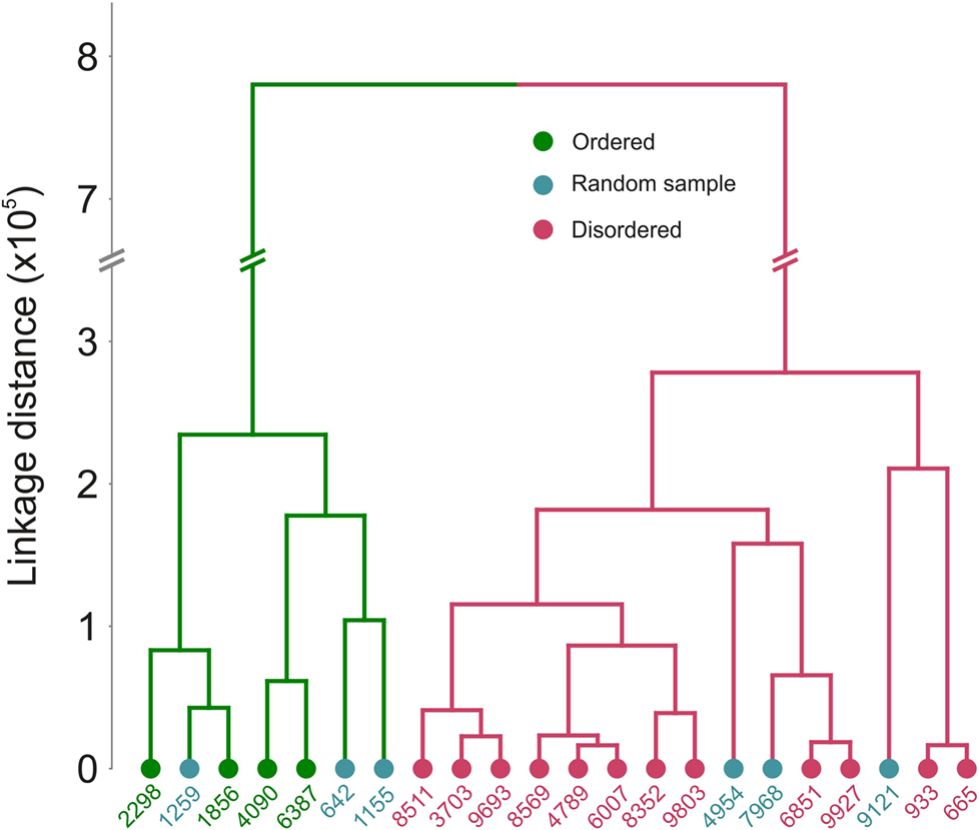
Hierarchical clustering of the electronic circular dichroism spectral data.

To compare the performance of bioinformatic predictors and the experimental sampling, the ECD spectra were subjected to hierarchical cluster analysis. Two dominant clusters clearly distinguished groups 1 and 3, the groups that were differentiated based on bioinformatic predictions. The ECD spectra of group 2 (randomly selected from the bioinformatics dataset) were evenly distributed between these major clusters (Fig. 4). Despite the small number of proteins sampled experimentally, the experimental data provide reasonable support for the power of bioinformatic predictors when applied to random sequences.

### Bioinformatic and experimental analyses highlight the evolutionary potential of random disordered sequences

**Figure 5:**
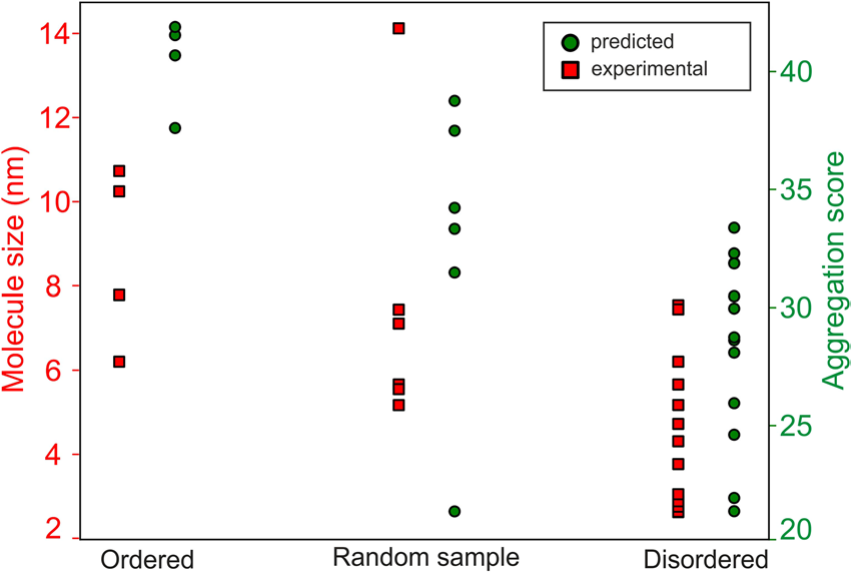
Aggregation propensity of selected never-born proteins. Results of dynamic light scattering analysis (left y-axis and red squares) and aggregation prediction using the ProA-RF algorithm (right y-axis and green circles) for the 22 experimentally sampled group 1–3 proteins.

**Figure 6:**
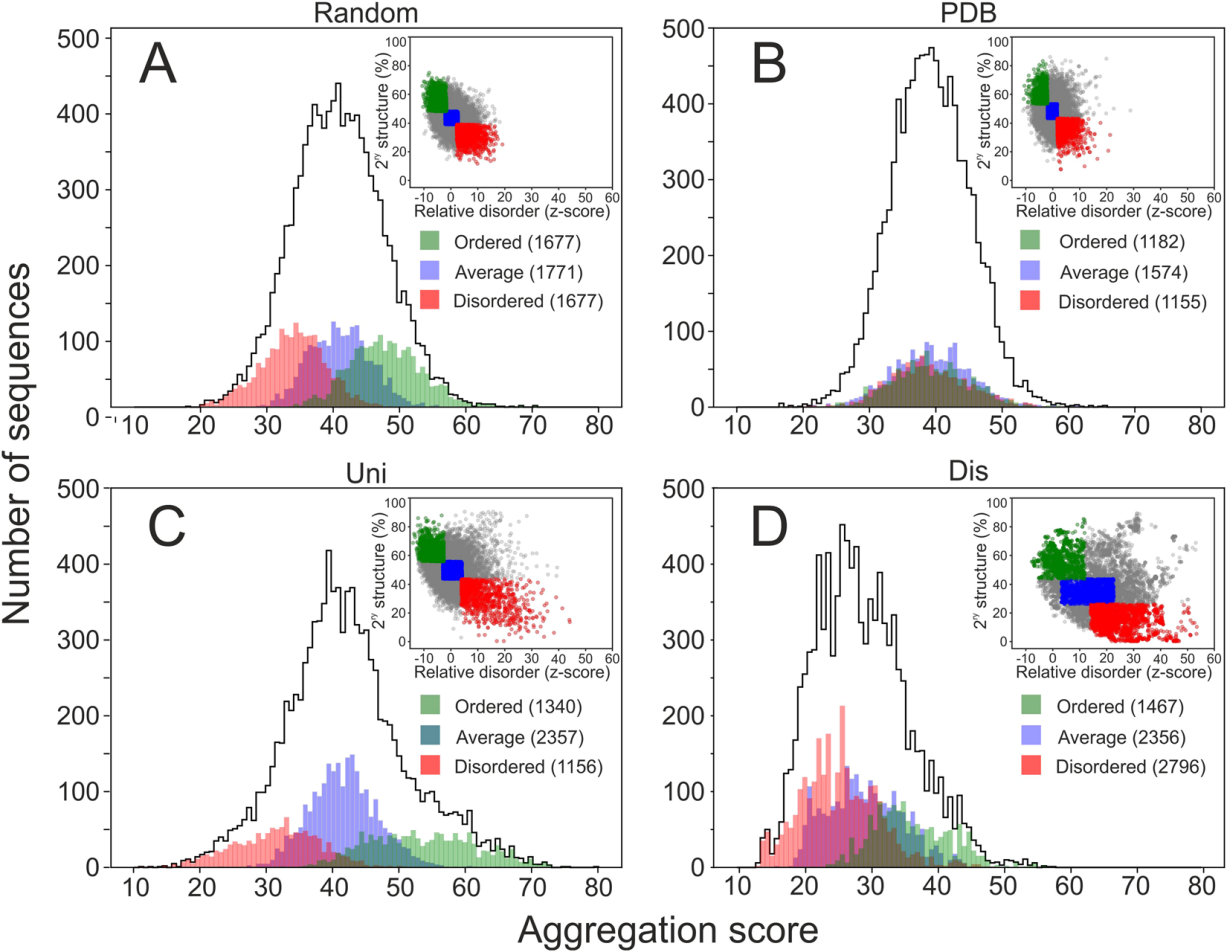
Aggregation propensity of the datasets depending on secondary structure analysis. Distributions of aggregation propensities for the entire random (**A**), PDB (**B**), Uni (**C**) and Dis (**D**) datasets showing the “ordered”, “average” and “disordered” subsets (<0.5 SD value, SD ± 0.5 SD, and >0.5 SD values, respectively, from mean values of predicted disorder and secondary structure). The top right corners graphically demonstrate the “ordered”, “average” and “disordered” selections from the secondary structure predictions (y-axis, total % of secondary structure content) and disorder (x-axis, z-score units) – equivalent to Fig. 2. Values in brackets for individual subsets in the legend indicate the population numbers.

Dynamic light scattering (DLS) experiments, expression/solubility profiles, and analysis of physicochemical properties relevant to aggregation using the ProA-RF algorithm all suggested that group 1 proteins (most structured) are prone to oligomerization and aggregation. In comparison, along with their better *E. coli* expression profile, group 3 proteins (most unstructured) form smaller particles in solution and do not tend to aggregate (Fig. 5)^21^. The same trend persisted for the entire random dataset when the ProA-RF algorithm was used to predict aggregation of the “ordered” and “disordered” segments of the dataset (Fig. 6A). Therefore, random sequences with higher structural content have a greater tendency to aggregate than those with less structural content.

It has long been suspected that random and IDP-like sequences would tend to aggregate and be toxic to cells. However, a recent computational study reported that random proteins do not have an increased aggregation propensity compared with existing proteins, which is in full accordance with our experimental and bioinformatic results^22^. Several studies have suggested that while natural IDP sequences were expected to be aggregation prone (because of the increased likelihood of exposing aggregation-prone residues to solvent at the absence of a hydrophobic core), it is not so probably as a result of strong anti-aggregation evolutionary pressure^23,24^. Our study demonstrates that low aggregation propensity is in fact a natural property also of random “disordered” sequences.

This trend is less pronounced for natural proteins in the Uni dataset (Fig. 6C) and completely absent for the PDB proteins (the PDB dataset contains proteins that were successfully expressed and structurally studied and therefore represents a biased sample of all extant proteins) (Fig. 6B). To better understand these differences, we performed sequence analysis of each library based on the structural content (ordered, average, and disordered). The ordered and disordered subsets deviated from the mean amino acid composition in the same fashion for each library (Fig. S4). As expected, the control Dis dataset generally deviated in amino acid composition, strengthening the trend observed for the “disordered” subsets of the other datasets. These deviations are in agreement with those observed in previous studies^25^. In addition, for natural proteins, these deviations may reflect a functional purpose. For example, according to ontology analyses (not shown), the “ordered” shoulder shown in Fig. 6C (aggregation score >60) is occupied mostly by membrane proteins. While amino acid composition generally affects the secondary structure content, it determines the aggregation propensity only in unevolved sequences. Evolutionary pressure can work with a given amino acid composition to minimize aggregation and/or prepare the protein for specific conditions, such as the membranous environment. Hypotheses that aggregation-prone sequences are disfavored by evolutionary selection and possible mechanisms for this phenomenon have been described in previous reports^23,26,27^.

In summary, random sequences are not significantly different from natural proteins in terms of secondary structure occurrence and overall aggregation properties. Random sequences with low structural content may actually represent advantageous origin points for further evolution into soluble functional proteins, as they are better tolerated *in vivo* and have lower aggregation scores than random sequences with structural content. This is consistent with recent studies reporting that random sequences are often bioactive and can even increase fitness *in vivo*, as well as work suggesting that non-coding DNA translation (one of the hypotheses about *de novo* gene birth) gives rise to highly disordered proteins^28,29^. It is therefore not surprising that structurally dynamic proteins are often encountered during protein-directed evolution experiments in which proteins are selected based on function (rather than structure) from sequence libraries, even if they are originally based on a structured scaffold^12,30^. If proto-proteins arise from random sequences with high structural content, they would likely be disfavored based on their natural physicochemical properties unless their aggregation properties are selected for. Our study provides rationale for this hypothesis on a protein-sequence-space scale.

## Methods

### Construction and bioinformatic analysis of in silico libraries

Using the composition statistics of the TOP8000 dataset, a library of 10^4^ random sequences (100-amino-acid randomized sequences both with and without additional 9 amino acids incorporated for recombinant expression, including an N-terminal methionine and C-terminal hexahistidine tag) was generated *in silico*. Each amino acid at a randomized position was picked randomly from the complete amino acid set with frequencies corresponding to the TOP8000 dataset. All positions in the random sequences were treated independently and without any correlation and additional constraints imposed with respect to the sequential neighbors, position in the sequence and the total composition. In parallel, we constructed three control libraries of 100/109-residue protein fragments from (i) the TOP8000 dataset of structured proteins deposited in the PDB database, (ii) the Uniprot sequence database, and (iii) the DisProt database of IDPs^18–20^.

The similarity of the random and the known proteins sequences was assessed by the BLAST method implemented in BLAST + 2.6.0 software package. The NCBI Protein Reference Sequences (Sep 18, 2017; 92 439 966 sequences) were employed as the reference database. The alignments were constructed using default parameters (BLOSUM62 similarity matrix, gap opening and extension penalty 11 and 1, respectively)^31,32^.

The secondary structure content was predicted using several methods – GOR4, Jnet, Predator, Simpa, and Psipred^33–37^. In addition, the libraries were analyzed by different protein disorder predictors (Disopred, DisEmbl, VSL2 and IUpred) and empirical indices predicting solubility (CVsol and Gravy)^38–43^.

To investigate the aggregation propensity of structurally distinct protein sequences, proteins of high (ordered), low (disordered), and average structure content were selected from the random, PDB, Uni, and Dis datasets. Secondary structure and protein disorder predictions for each sequence were combined with the intention of reducing the false positive rate of individual predictors. Sequence selection into the three groups was based on the following criteria:

**Table:** 

	**Ordered**	**Disordered**	**Average**
**Secondary structure**	$S{S}_{cont}\ge {\mu }_{SS}+\frac{1}{2}\cdot {\sigma }_{SS}$	$S{S}_{cont}\le {\mu }_{SS}-\frac{1}{2}\cdot {\sigma }_{SS}$	${\mu }_{SS}+\frac{1}{2}\cdot {\sigma }_{SS}\ge {\boldsymbol{S}}{{\boldsymbol{S}}}_{{\boldsymbol{cont}}}\ge {\mu }_{SS}-\frac{1}{2}\cdot {\sigma }_{SS}$
**Disorder**	$Disorder\le {\mu }_{dis}-\frac{1}{2}\cdot {\sigma }_{dis}$	$Disorder\ge {\mu }_{dis}+\frac{1}{2}\cdot {\sigma }_{dis}$	${\mu }_{dis}+\frac{1}{2}\cdot {\sigma }_{dis}\ge {\boldsymbol{Disorder}}\ge {\mu }_{dis}-\frac{1}{2}\cdot {\sigma }_{dis}$

SS_cont_ is the total secondary structure content of the sequence; Disorder is the relative disorder score of the sequence; *μ*_*SS*_ and *μ*_*dis*_ are the mean values of total secondary structure content and relative disorder score distributions, respectively; and *σ*_*SS*_ and *σ*_*dis*_ are the standard deviations for those distributions.

Per-residue aggregation scores were generated with the ProA predictor. The final aggregation score for each sequence was obtained by summing all per-residue scores from the ProA output.

### Experimental screening of the in silico library

#### Protein expression and solubility analysis

DNA sequences encoding the 3 × 15 selected proteins were codon-optimized for *E. coli* expression and synthesized by Thermo Fisher Scientific, USA. The DNA sequences were subcloned into the pET24a plasmid using *NdeI/XhoI* restriction sites, and the resulting proteins had an additional Met residue at the N-terminus and a Leu, Glu, and 6 × His-tag at the C-terminus (equivalent to the sequences used for bioinformatics analyses controls). The proteins were expressed in 5 mL cultures of *E*. *coli* BL21 (DE3) for 5 h with 0.5 mM IPTG at 30 °C. The cells were harvested, and pellets were resuspended in 0.5 mL B-PER reagent (Thermo Fisher, USA) supplemented with 5 U/mL benzonase and 100 µg/mL lysozyme. The lysate was centrifuged at 13,000 × g for 10 min at 4 °C, and the supernatant (soluble fraction) was separated from the pellet (insoluble fraction). The pellet was resuspended in 7.5 mL SDS-PAGE sample buffer, and 10 µL soluble fraction and 6 µL insoluble fraction were analyzed by 18% SDS-PAGE. As a control, bacterial pellet from 1 mL pre-induction culture was resuspended in 1 mL sample buffer, and 30 µL of this sample was analyzed in parallel. After electroblotting onto a nitrocellulose membrane, proteins of interest were specifically detected with an anti-His-tag iBody (a synthetic antibody mimetic, present at 5 nM concentration) overnight at 4 °C^44^. The conjugate carries the Cy7.5 fluorophore, which was detected using an Odyssey CLx Imager (LI-COR)^44^.

The identities of all expressed proteins were verified using LC-MS following in-gel tryptic digest according to standard procedures^45^.

#### Large-scale expression and purification of selected proteins

Larger-scale expression and purification were attempted for all proteins that expressed in a soluble form. Some proteins that were not soluble in the initial analysis were also overexpressed on a larger scale after further optimization of the expression conditions to solubilize them (such as decreasing the expression temperature). Briefly, proteins were expressed in 0.2–4 L of LB medium for 4–12 h, typically with 0.2 mM IPTG at 20–37 °C, depending on the individual optimal conditions. The bacterial pellets were resuspended in 50 mM phosphate buffer, 30 mM NaCl, 1 mM 2-mercaptoethanol, pH 8, and sonicated 5 × 30 s on ice prior to centrifugation at 20,000 × g for 30 min at 4 °C. The supernatant was subjected to purification on Talon matrix (Clontech, USA) using a gravity-flow arrangement. The eluted fractions (in 50 mM phosphate buffer, 30 mM NaCl, 250 mM imidazole, 1 mM 2-mercaptoethanol, pH 8) were dialyzed thoroughly into 10 mM Tris, 10 mM NaCl, 1 mM TCEP, pH 8, and concentrated to approximately 1 mg/mL before further characterization. Where preliminary DLS measurement suggested a mixture of aggregated and lower-oligomeric species, gel filtration chromatography was used to isolate the lower oligomeric form. Only 22 proteins were purified in sufficient quantity and stability to allow downstream characterization.

#### Biophysical characterization of selected proteins

Prior to analysis, the identities and molecular weights of purified proteins were confirmed by mass spectrometry. In addition, precise protein concentrations were determined by amino acid analysis using a Biochrom 30 + Series Amino Acid Analyser (Biochrom, UK).

The same protein preparations were used for ECD and DLS measurements. ECD spectra were collected using a Jasco 815 spectrometer (Japan) in the 185–300 nm spectral range using a 0.02 cm cylindrical quartz cell. The experimental setup was as follows: 0.1 nm step resolution, 5 nm/min scanning speed, 16 s response time, and 1 nm spectral band width. After baseline correction, the spectra were expressed as molar ellipticity per residue θ (deg·cm^2^·dmol^−1^). If needed, samples were diluted in 10 mM Tris, 10 mM NaCl, 1 mM TCEP, pH 8. To collect ECD spectra with co-solvents, the samples were diluted to reach the final concentrations of 0.4 M GuHCl and 50% (v/v) TFE.

The ECD spectra of individual proteins were subjected to hierarchical cluster analysis using the Euclidean distance and Ward linkage algorithms in the MATLAB environment (MathWorks, USA).

One dimensional hydrogen NMR spectra were acquired at 25 °C on 850 MHz Bruker Avance III spectrometer equipped with a triple-resonance (15 N/13 C/1 H) cryoprobe. The sample volume was 0.35 ml.

Prior to DLS measurement, the protein samples were centrifuged at 20,000 × g for 30 min at 4 °C. To completely remove dust particles, the samples were immediately filtered using 0.1 μm Ultrafree^®^-MC centrifugation filters (Millipore, USA). The measurements were performed at 20 °C using a Zetasizer Nano ZS instrument (Malvern Instruments, Great Britain) equipped with an internal 633 nm He-Ne laser. Proteins were measured in a 3 × 3 mm quartz cuvette (internal volume of 40 μL). The results were processed using the original Zetasizer 6.2 Malvern Instruments software (Great Britain).

## Electronic supplementary material

Supplementary information
