# Supplementary material for: Random protein sequences can form defined secondary structures and are well-tolerated in vivo
Source: Sci Rep. 2017 Nov 13;7:15449. doi: 10.1038/s41598-017-15635-8 (PMC5684393; doi:10.1038/s41598-017-15635-8)
Supplement: Supplementary file 1 — Supplementary information [file 41598_2017_15635_MOESM1_ESM.pdf]

Random protein sequences are potent with secondary structure and well tolerated *in vivo*

Vyacheslav Tretyachenko [1,2], Jiří Vymětal [1,2], Lucie Bednárová [2], Vladimír Kopecký Jr. [3], Kateřina Hofbauerová [3], Helena Jindrová [1,2], Martin Hubálek [2], Radko Souček [2], Jan Konvalinka [1,2], Jiří Vondrášek [2], and Klára Hlouchová [1,2] *

[1]*Department of Biochemistry, Faculty of Science, Charles University, Hlavova 2030, 128 00 Prague 2, Czech Republic*
[2]*Institute of Organic Chemistry and Biochemistry, The Czech Academy of Sciences, Flemingovo náměstí 2, 166 10 Prague 6, Czech Republic*
[3]*Institute of Physics, Faculty of Mathematics and Physics, Charles University, Ke Karlovu 5, 121 16 Prague 2, Czech Republic*

* For correspondence: klara.hlouchova@natur.cuni.cz

Supplementary information

Table S1. Sequences of proteins from experimental groups 1-3 and the results of their BLAST analysis

ID (random sequence)	Random sequence	NCBI Reference Protein Sequence ID	Description of the protein	E value of the match	Length of the match	Identities	Positives	Gaps
Group 1								
8836	MGAGLAQFQIDPLYRMAVDDLPKDTIWGNIRKAFEEAFLER TYSANKYDLKALKHLTGKQYEDVICAHKRVLDYAITRDEYYL AGDAANIFRGLVSARKAELEHHHHHH	WP_023721735.1	hypothetical protein [Mesorhizobium sp. LSHC420B00]	0.1	84	28	44	4
3027	MMMFSALKYVDSYAHYEFKRIDDDHEDSFLKSAATRTGIRE DRALLKVGAAVLEYSLSITEVAGLRVQYRTEKDPWTDLLNL QAMRTSYNHAWYPQELVKVLEHHHHHH	No significant match						
1856	MYQIEKADFTFDVRRRTAATDIENHAFNMVWLQSWCDVSII KRTLDAYDEAYDAAFQRLKPAEWAIDDWVASIQRRRRHYVA YNLSKIKLPVRLEKLSGTTLEHHHHHH	WP_036380662.1	Na/Pi cotransporter family protein [Mogibacterium timidum]	2.0	49	17	29	0
1441	MKKKAGKQPGDECNFRWKMFTNTMVYRRNLMTAGFYGLE AAREDYLKVNRMIKEYLERYALSTQRLFHQLMTNWEFIKRGI CLDTYLRDTRILDYETIVYILEHHHHHH	No significant match						
6387	MIEHCYSKTVYYNLEQEKYDLEVTHIEGWMRAGRKDLADN LLEDSGHVFIPEVALQENHYREVHAKIGDAEMRVYKRELFP EPQIVEVLETPSQLFFAEIELEHHHHHH	WP_088273924.1	flagellar biosynthesis protein FlaG [Pseudomonas aestusnigri]	4.0	36	16	25	0
1687	MRQYFLDLKMYICELGFLHDARQNGMGETYDELNQRQWR EADVYILMDNGSLLGDFSHKKYILARYANIRTAEEMVGGSFT IAQFAKSVEFGGDHARKLSALEHHHHHH	WP_045055488.1	hypothetical protein [Aliterella atlantica]	3.4	81	27	42	10
8635	MPFLLFGGTLTIEKVAILKPTLMFHATLLEIKGLLLRNLPHNDR ATAWQFLYSENLLNASRLQDTDEFFKYSKVESSGLYQEVRD KATNRDQFDKLNAYHELEHHHHHH	WP_067658158.1	S9 family peptidase [Ferrimonas marina]	1.8	63	20	35	7
3451	MWSKYCSYMTSIWADRSEAADNHQWFSLDIHHIGGWQPK RGEKFEHVVRDLVTRGEARLIYIARSVVEVAGIYTRRYDVTA SLSQLEDAELEDLDTLEYALLEHHHHHH	WP_059176866.1	dephospho-CoA kinase [Thermodesulfovibrio aggregans]	2.1	57	17	33	1

ID (random sequence)	Random sequence	NCBI Reference Protein Sequence ID	Description of the protein	E value of the match	Length of the match	Identities	Positives	Gaps
6620	MFITIRQEDQVGERRFIPMLIFRKEAGNGLTDDYLLAYWSRSIEGINEDWNFGEINQILERGRDRERSILSNNKVAQLIYAIMWWLRIAELAPAQISKLKDLEHHHHHH	WP_067751631.1	SusC/RagA family TonB-linked outer membrane protein [Niabella ginsenosidivorans]	5.4	45	19	25	2
8600	MKVDMELRLNPLHGKMSQVVFFADLVAQYKFRGITRRAKYAKKGMALYGIKNHFAEENLCYEIKGLSLHKYEALNSKKRLYLLSKAIWARNMIKKDLDRQPLEHHHHHH	XP_020969492.1	(3S,6E)-nerolidol synthase 1 isoform X2 [Arachis ipaensis]	3.7	46	16	27	1
4712	MFTNRFDDYTLDTVKRVFEYTITYLRVGPGFRTMAYTNDVEYDHGADHPVGGRQGVDSPVRPQEGSMWNCRNYLVYVQGNTLVQIEGYFVIAKPLDQDWDVLEHHHHHH	XP_017483684.1	PREDICTED: probable ubiquitin-conjugating enzyme E2 C [Rhagoletis zephyria]	1.8	35	14	20	0
2423	MTKYRAYVLTANIGYALYERADATVHQYHYQSEPHWNQQVIGNNKPYVKVLVVVINDEVYSTDKDSHLQEMTYIYTPILNPFQSDELTWVKYIRDNYGGGDLEHHHHHH	XP_007510405.1	unknown protein [Bathycoccus prasinos]	1.2	54	18	29	0
7144	MITMPLLAIEDNDLFYVRTYPHLFCVGMYNQDKGKFNYVDYEALRVVNKDKTEAKLTWTSHTHGLEALHTGASRPLFAKPYEGSGHQHFIDNLYPPHLEVALEHHHHHH	XP_022082075.1	LOW QUALITY PROTEIN: uncharacterized protein LOC110974603 [Acanthaster planci]	5.5	45	14	23	0
4090	MVERDKPPNIWVYDAEPLQQGGIVWVHLAALYCANVDDYAPQDHLDITMYGFDHQKTNILSFEDESVNAQSYWQYGIIFVKSHWGEDLQGAIVESWRDSRSLEHHHHHH	WP_075092519.1	Tet(A)/Tet(B)/Tet(C) family tetracycline efflux MFS transporter [Planctomyces sp. SH-PL14]	6.3	23	11	16	0
2298	MKWYGRGREDFGSPDVDVEKNCEGEVIYGTSQELYSNVVFDWWAGISEQPTIFIGSLTTPNTKDDMLWYRNDAKNPGHSILYNLINDYWEATEVSGIGNVVLEHHHHHH	No significant match						
Group 2								

ID (random sequence)	Random sequence	NCBI Reference Protein Sequence ID	Description of the protein	E value of the match	Length of the match	Identities	Positives	Gaps
5872	MCHSIDVLQQQRKVAQPSLPVLKVLRHWKLDDLYCDATWIIEMLESPFFKNGTTHNIELLPRILPTQHIHVASLLPRGARPKIVLESNDIDQGWADESEEGLEHHHHHH	WP_020569377.1	SMI1/KNR4 family protein [Lewinella persica]	0.4	65	19	39	6
5493	MNTLEIIIGYERAYIKLILLYNQLYVEMEGLSGLPNYVVARWFKLSRSIFLAPPPRARKAAESTWQHADFTGSDTIVPEETLLAAIDNGVYLEVPELLTFELEHHHHHH	XP_009497541.1	hypothetical protein H696_05432 [Fonticula alba]	0.62	60	24	30	1
2920	MMVDVDYPARQHLHYLFIGPFENTAGNSLIKGYVGYQVRIGRRPAETPQCTAVKMHDAGAKHESPLGAERNSVLDHGSTISKITTLFGPTADTRQMGSEEALEHHHHHH	XP_008619127.1	hypothetical protein SDRG_14753 [Saprolegnia diclina VS20]	2.0	74	25	34	4
1155	MDQTKCSSAAVSVAAHKTTNKQDADSDYFKNSTHLTPDPRVLVRNEMDTMFEVNSLILYEHSAALTGAQISKSMKDLCTGLEFKVYPPDMIIDAVQRHHVLLEHHHHHH	XP_020883070.1	LOW QUALITY PROTEIN: serine carboxypeptidase-like 9 [Arabidopsis lyrata subsp. lyrata]	8.6	62	21	27	11
4954	MRVIGLSESPFDPAPKFPAASLAVGPVDGNKVPDLLLQAVLALMHTSSLKNSLNIANVVRANITPRRSQPMAKRPSILFRHSSLERDLIIVWVNDSIKDEALEHHHHHH	WP_087015815.1	hypothetical protein [Leucobacter sp. 7(1)]	2.5	49	19	28	0
642	MNEMAGSGAGSYSKYVNENPVTKPMAIMSAKEVWLEYEAWDPVRNQLPIYRRDDFWYYPDCQTKVFRHPWERESAIYLPMTDPTAVDRISAATDDSFTNLTLEHHHHHH	WP_066423087.1	DUF1684 domain-containing protein [Flavobacteriaceae bacterium CP2B]	2.3	94	32	42	16
7968	MVLDMPAAQKGPLQMAGTELAAEEVKNGGQSTGHSLRSLRLDVAEGPSILDRLESHRETLHDKMEAAHELQGKKDWSLMNQYSDRGADSEHPFLKGEVTKYLEHHHHHH	WP_088904803.1	beta-glucosidase [Shewanella marisflavi]	4.2	36	16	23	0
9121	MEDPTGVPFHHKKSHNMRKVETVNYGHKGCLVITDETNSLGVPGVDLEGLQGAGQLAMYKPTHGIEMARAESELIARRKEHGKMAFIIVHRELIPEEDNRFLEHHHHHH	WP_030437445.1	beta-glucuronidase [Actinoplanes subtropicus]	0.75	92	29	44	5
31	MGGGASGFVGPQETADLNRYRYVITQRKKDNEERKSVDLYAFILDTSYGVEEKKNEYNAIDARPGLPWNHANVGVIVVWSATNSHDPKMILGDRARARRTVLEHHHHHH	WP_027672490.1	hypothetical protein [Rheinheimera baltica]	0.67	105	31	47	7

ID (random sequence)	Random sequence	NCBI Reference Protein Sequence ID	Description of the protein	E value of the match	Length of the match	Identities	Positives	Gaps
7106	MHLVQTGTAIKELIGTLVVICHFNWLIAPELEGLHCDFPERFAEMWRVGYGGHHLIFLARTTQYEDGVPGLEHVVIGLKQPEGGGYCRPNVSGKVAAYRFVLEHHHHHH	WP_045796070.1	DeoR family transcriptional regulator [Acinetobacter indicus]	1.8	54	18	29	3
7530	MDKGGEAEQVTPMNKEKGSEGIGVMSHAHKNEKENFAIVGIINDSLVVPAHETLVITCAYGLSLVAKARGIKGVPKVVFTMSDKLLSAIGAGMPTKVGTWPLEHHHHHH	No significant match						
27	MHSAWDLVPQLRSPHLSCINMRVIKSWDLTRMVEFLDYQNTKADGFNHKMNRATEAEIATDSKARGYVFKLARTGMNSLVADLGAGKATLGHAPMKAVGLRLEHHHHHH	XP_020477136.1	protein disulfide-isomerase A3-like isoform X1 [Monopterus albus]	1.9	96	28	45	7
1259	MSLGYEASEDGTRVEAIKDGGRDGHMDIEAGGCGYWLFDHKDHRYAEHPMINTRDLDAQMFAMFAIFYEQDTIGFPDNPWSLIILPAVSYMSMMEFAPAPTLEHHHHHH	WP_010040813.1	hypothetical protein [Gemmata obscuriglobus]	7.2	103	26	49	8
1684	MFWKHLTTKQAHEKETSNAMLRHAIPTAIACGIPPQFTQKTGLVLKRSPTCNMHDPAQCTRIKMFEVLRIWDVAMKMDYSGAKYYESYQCESTWGMPGAFLLEHHHHHH	XP_013415555.1	PREDICTED: acetylcholinesterase-like [Lingula anatina]	3.9	69	21	32	2
2436	MWRQHIMSIFLDRQGLDISLYDRSTEAMAQKGSSKNRGEYGHHQPTNVGFESRQAHGRYQGEFHSRVVSMFQQLIVVEEYKQYDGHTVTTYAANKEVVTEELEHHHHHH	WP_087624986.1	FAD-binding oxidoreductase [Aeromicrobium sp. PE09-221]	3.4	61	17	32	2
Group 3								
665	MATKGADHGLAAPQPHAKWDTQIPAEGADREHRSGGGNERRFYNEGAKHAQATWAIPDEPAFHLQPAVGEGATTDQAGSLEDQWVRSLNNNDVDPTQADETLEHHHHHH	WP_005373007.1	ABC transporter permease [Methylomicrobium album]	2.3	70	24	33	4

ID (random sequence)	Random sequence	NCBI Reference Protein Sequence ID	Description of the protein	E value of the match	Length of the match	Identities	Positives	Gaps
8667	MPQSLACAGTATRESQNDQLLDGHQPETYLDRMFPEELDE<br>IDGVIPNYDMEWGKKSGLEVSEKCFDPWFNYPTYEETPSD<br>MSGPFKNALMKYRPTQNARPDLEHHHHHH	WP_073962213.1	aminoacetone oxidase family FAD-binding enzyme [[Ruminococcus] gnavus]	2.9	53	16	27	4
3703	MSLYKFGQRRAVDPLPRQCQRDKDYDAFIGGEQNCDNELS<br>KSFPIVVMSVFLYDPTYNVDSEAQDNKLDHHGSEPTHGDTP<br>TTSEDTRPGSDRVMRDVPQTLEHHHHHH	WP_058364961.1	hypothetical protein [Haloparvum sedimenti]	0.073	49	18	27	1
933	MVREIDDKTISDYLARGADEGTTAYSLKIPTDKCLFAPTKKHL<br>HGGDKSQEADPPTKSPMVEHQFGHEPDFPSCREPEDYPG<br>SPLVELTGLNRLTQEPNEELEHHHHHH	XP_019635581.1	PREDICTED: actin-binding LIM protein 1-like isoform X6 [Branchiostoma belcheri]	7.3	72	25	35	8
9927	MHPAEVSFSGGAPNNESKWDNRHYVQAESGEDHETGVHL<br>GDFDEYLSLQAGPRLPMPELSGHWGSQCLNCDKGGKKKA<br>NIVPSPEDVSKDVKSYEGFEADRLEHHHHHH	WP_072625341.1	NADPH-dependent 2,4-dienoyl-CoA reductase [Janibacter indicus]	2.3	47	18	24	0
8352	MNREAEWLREPLPKYYDFAGTYNTRMTEWGDKALTTVVV<br>GTLYQVEEPPGLTPASPMQHGACNAYNSELGFTQNEGALK<br>QEMPVIPGGGSDPERRGLADPDLEHHHHHH	WP_058353727.1	MarR family transcriptional regulator [Acetivibrio ethanolgignens]	2.5	108	26	48	15
5702	MKKSQKTGKFAPVTQQFPQASHCTVDKDYDHGYDGTNKP<br>EELMPAFFEINPEHDSAPALFAPDTMSSYEHLDTQGSHTVPI<br>SWGDSPEISFRAYEDIMFLDLEHHHHHH	XP_020873303.1	glycerophosphodiester phosphodiesterase GDPDL3 [Arabidopsis lyrata subsp. lyrata]	6.0	67	22	34	12
8569	MPQGVDNLVISWQAGEEGKNAGFIMGYLGVVGIDALDYQN<br>DPAQHYNDAQHDKKENYYHKGNPKPAELDAEGLGSEEPAL<br>PDHSPKVEELSIPDMYRPEQYLEHHHHHH	WP_066510348.1	nucleoside 2-deoxyribosyltransferase [Bradyrhizobium sp. BR 10303]	2.7	77	26	37	10

ID (random sequence)	Random sequence	NCBI Reference Protein Sequence ID	Description of the protein	E value of the match	Length of the match	Identities	Positives	Gaps
8511	MATDHIDDPGILAAFPAGTCLLDDKGKTSGATFAPASEEDIEGNPTGAAGDNLAYGEPTAYYSFINQPKEISGRQSSESSQHERVQMAERDYDDFTDQPTGLEHHHHHH	WP_072976053.1	cobalt-precorrin-2 C(20)-methyltransferase [Tissierella praeacuta]	0.33	74	26	34	9
9693	MMNGERSLIPDSMKSISAVRLIICGLIKPATAGLKEVDMHVWPNPTSLAGHSVSLYSSGKQISNLAFGDEESPNRERETAPADEDVIPDAHDTSDSLDGEHLEHHHHHH	WP_078964881.1	haloacid dehalogenase [Streptomyces aureocirculatus]	1.0	80	30	37	12
6007	MGHPELEEHDVQTNKEHHTNSQINLSTEVDLEPMGHKIDYPLANYVSPRNDYLPIFTISFMGFGGGRATPKETNVIAGTDPYKFTVSQSEDDLHGIADPRLLEHHHHHH	WP_070493048.1	TolC family protein [Neisseria sp. HMSC15C08]	4.0	46	19	24	0
4680	MAEEFLIREHGEGGSITYVLEGFYGPHPSCGQDVLPNYNMAKESYDTGTAEDDFEDRDTGSGAGWMRHDAGKDHKSESPRNALNYLYIYTNFRSLDRRGHVLEHHHHHH	No significant match						
6851	MLGIVEETEHTGREGERDLDKSLQFSDGLFMEVTVDQANKLGATKASTCTKEDGPSRCRSQHTITNLIEFSDSVDKTEKTDKEGRTGPMNSELVGGQEDEDLEHHHHHH	WP_046353406.1	GNAT family N-acetyltransferase [Janthinobacterium sp. B9-8]	4.6	39	15	23	0
4789	MQGDFNPEPGRSSNTSPRRYRIDEPGIRKSGAPLDRDVSDEASSHVNYPAVSEIPDFEDDFTGDIPIGPADKQLVETDGHNGQLDLGLNWDDIASLIIFKILEHHHHHH	XP_015747442.1	PREDICTED: uncharacterized protein LOC107327207 [Acropora digitifera]	3.2	94	26	41	8
9803	MEVGHKAFPRSMEDTSDKVQLIAPGEINMFTSGYEDNPMVMVKNNFEGDTKFHSDDVPIENMDDEAEDAPYLNVGNGRLVYTTDPDGLDATLETPGYLEDGLEHHHHHH	XP_021744940.1	uncharacterized protein LOC110710902 [Chenopodium quinoa]	0.68	70	26	35	6

Table S2. Average secondary structure content of the random, PDB, Uni and Dis datasets based on the GOR4, Jnet, Predator, Simpa and Psipred predictors

Dataset	Secondary structure	Mean secondary structure (%)	Standard deviation
Random	α-helix	0.28	0.12
	β-sheet	0.16	0.07
	Total	0.44	0.09
PDB	α-helix	0.30	0.15
	β-sheet	0.17	0.08
	Total	0.47	0.10
Uni	α-helix	0.34	0.17
	β-sheet	0.15	0.09
	Total	0.49	0.13
Dis	α-helix	0.26	0.18
	β-sheet	0.09	0.07
	Total	0.35	0.17

Table S3. Correlation table of the secondary structure prediction in Table S1

	GOR	*Jnet*	*PREDATOR*	*PSIPRED*	*Simpa*
GOR	1				
Jnet	0.66	1			
PREDATOR	0.55	0.73	1		
PSIPRED	0.56	0.72	0.70	1	
Simpa	0.61	0.66	0.69	0.74	1

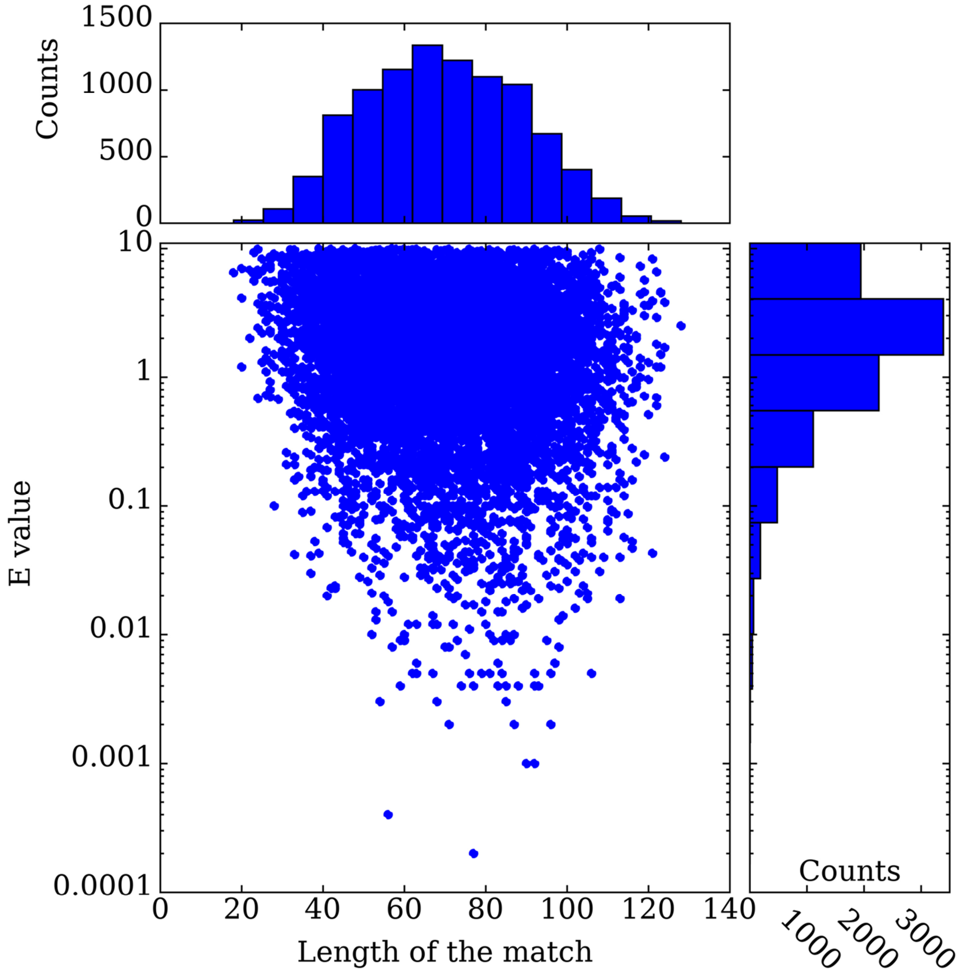

Figure S1: BLAST analysis of the Random dataset. Only the best match for each random sequence is plotted as function of the length of the alignment and the corresponding E (expectation) value. Expectation value measures the significance of the match, the lower the more significant match is. The side panels (top and right) show histograms projected on the particular axes.

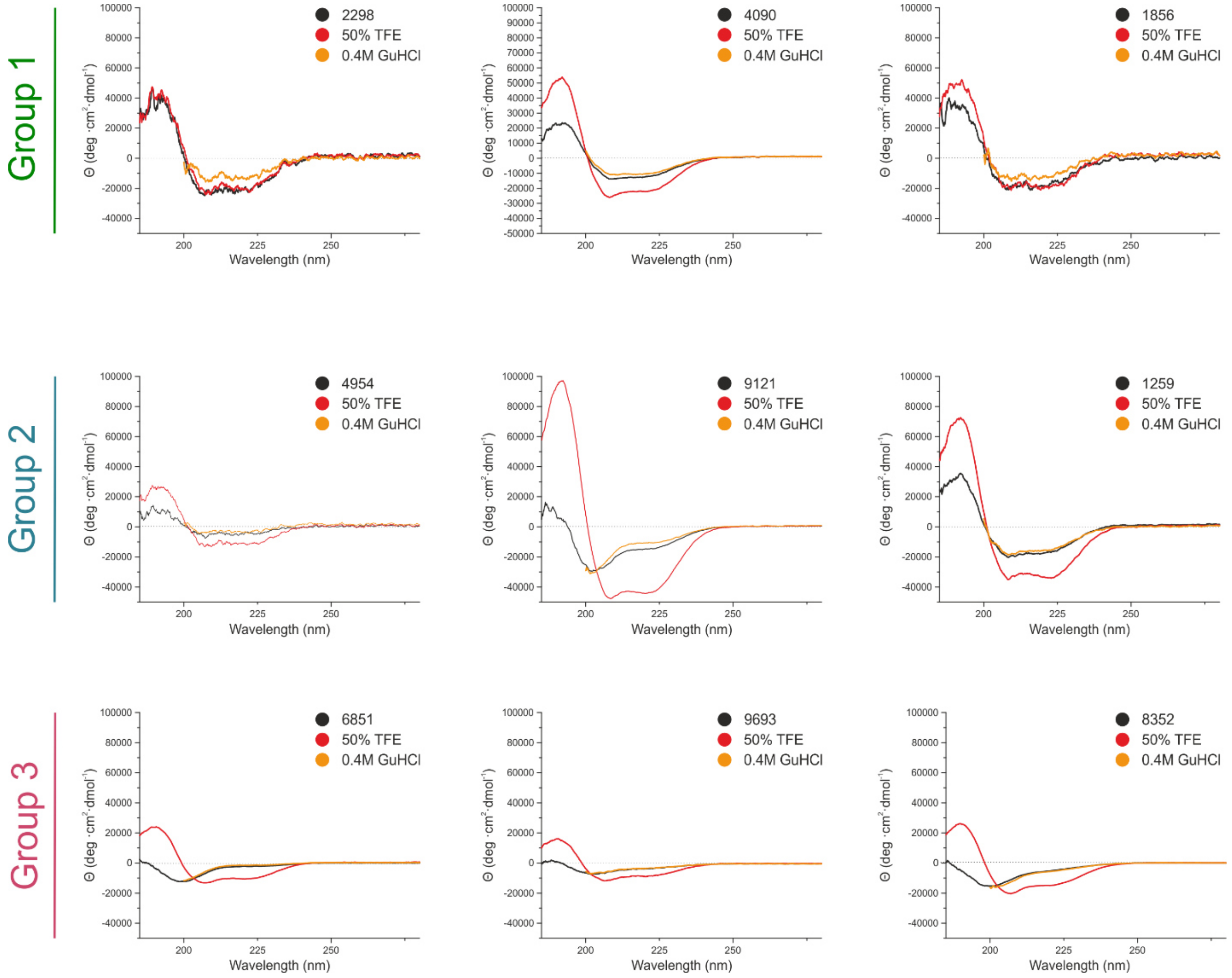

Figure S2: Example CD spectra of group 1-3 with co-solvents (TFE – trifluoroethanol, GuHCl – guanidium hydrochloride)

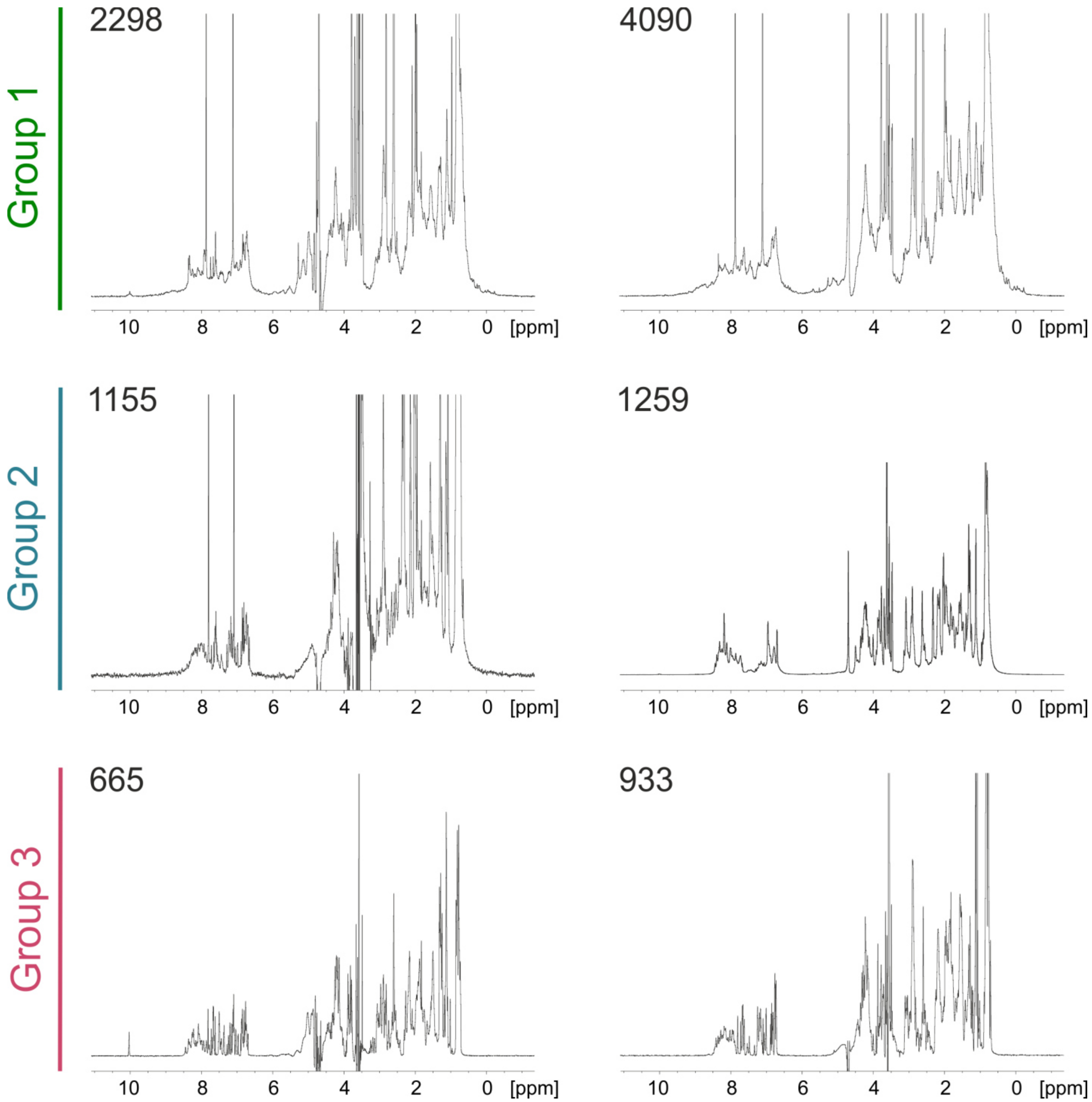

Figure S3: ^{1}H-NMR spectra of representative group 1-3 proteins

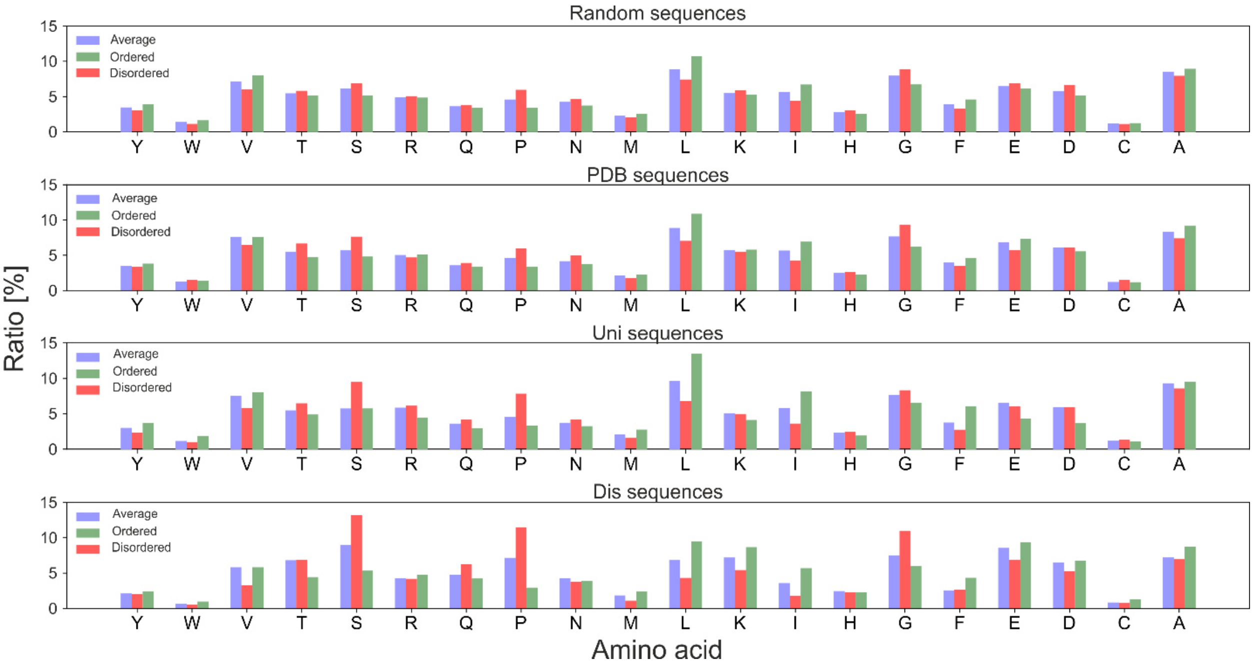

Figure S4: Analysis of amino acid composition for sequences belonging to the average, ordered and disordered subsets of individual datasets.
